# SIMPLE Technique of Laparoscopic Nephrectomy for Ectopic Nonfunctioning Pelvic Kidney Secondary to Pelviureteric Junction Obstruction: A Feasible and Safe Technique

**DOI:** 10.1155/2014/367246

**Published:** 2014-07-21

**Authors:** Santosh Kumar, Kalpesh Mahesh Parmar, Sriharsha Shankaregowda Ajjoor, Nitin Garg, Kumar Jayant, Shrawan Kumar Singh

**Affiliations:** Department of Urology, PGIMER, Chandigarh 160012, India

## Abstract

Ectopic kidneys are rare developmental anomalies. Anomalous blood supply of the pelvic ectopic kidneys poses a problem for a minimally invasive surgery. Although laparoscopic nephrectomies have been described for symptomatic nonfunctioning pelvic ectopic kidney, this is the first case report that highlights the safety and feasibility of SIMPLE technique of laparoscopic nephrectomy in a pelvic kidney.

## 1. Introduction

Renal pelvic ectopia is a rare congenital anomaly with a reported incidence of 1 in 3000 autopsies. Ureteropelvic junction obstruction (UPJO) occurs in 22% to 37% of ectopic kidneys. Majority of cases remain asymptomatic and are diagnosed incidentally. Surgical intervention is indicated in symptomatic cases and in patients with obstructed drainage. Although laparoscopic nephrectomy for nonfunctioning pelvic kidney has been described in the literature, we herein describe single incision multiple port laparoendoscopic surgery (SIMPLE) technique of laparoscopic nephrectomy in a 43-year-old male with ectopic nonfunctioning pelvic kidney.

## 2. Case Presentation

**Figure 1:**
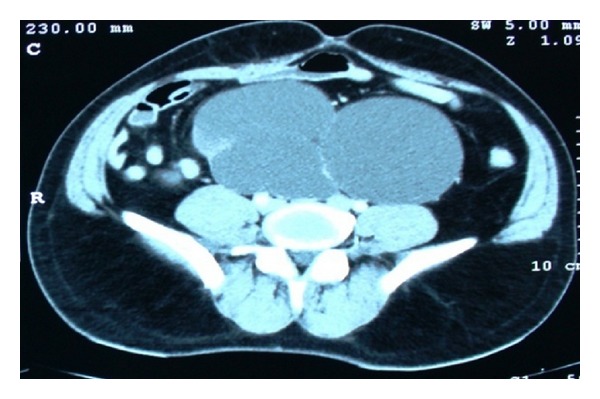
Axial CECT abdomen image showing grossly dilated right renal pelvic kidney with thinned out parenchyma.

**Figure 2:**
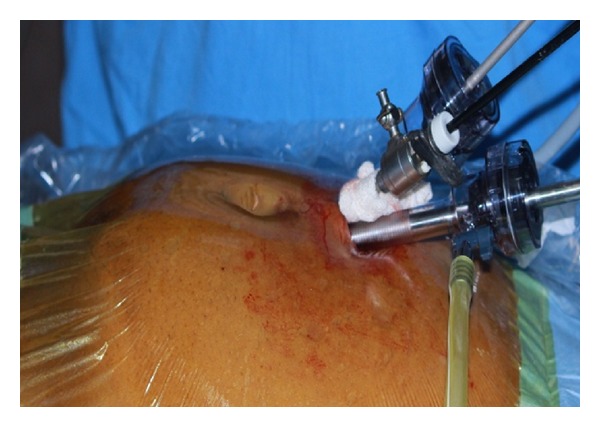
Single incision multiple port placed supra umbilical region.

**Figure 3:**
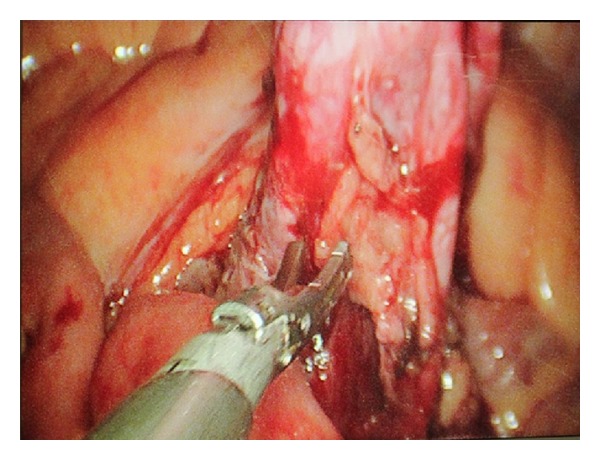
Renal pelvis dissection.

**Figure 4:**
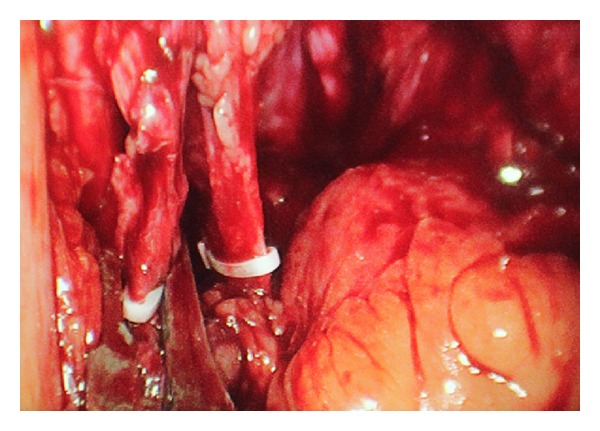
Clipping of the renal vessels.

A 53-year-old male patient presented to our institute with complaints of right flank pain of one year duration. General physical examination was unremarkable. On per abdomen examination, a vague lump was palpable in umbilical and hypogastric quadrant. USG abdomen revealed an ectopic right pelvic kidney with gross hydronephrosis with thinned out renal cortex. Routine hematological and biochemistry profile was normal. Renal dynamic scan reported nonfunctioning right kidney. To know the renal vascular anatomy, CECT abdomen with CT angiography was done which revealed grossly hydronephrotic right ectopic kidney placed in the pelvis with thinned out cortex (Figure 1). There was single left renal artery arising from abdominal aorta at L1 level. The ectopic pelvic kidney was supplied by 2 arteries, one is from the abdominal aorta just proximal to its bifurcation and the other one is from the left common iliac artery. The patient underwent laparoscopic nephrectomy by SIMPLE technique and was operated under general anesthesia. Cystoscopy was done initially which showed bilateral ureteric orifice in normal position and bilateral ureteric catheters were placed. The right ureteric catheter was crossing the midline on left side and was placed in the renal pelvis of the right pelvic kidney. The patient was positioned in supine trendelenburg position. The patient was adequately supported and strapped, and all the pressure points were protected. Veress needle was used to create pneumoperitoneum. 2.5 cm incision was made in the umbilicus. We used conventional laparoscopic instruments during the surgery. A 10 mm port was inserted at the umbilicus, and the other 10 mm and 5 mm ports were placed adjacent to that in the same incision (Figure 2). Posterior peritoneum over the pelvis was incised and right pelvic kidney was localized. The ureter was localized over the psoas muscle and dissected till pelviureteric junction. The renal pelvis was dilated which helped in dissection around the kidney (Figure 3). Multiple vessels were present over the renal pelvis. The renal pelvis was decompressed with an externally placed needle. The renal vessels were dissected and isolated. The vessels were clipped and cut (Figure 4). The kidney was mobilized all around and the final specimen was removed from the umbilical port site. Hemostasis was achieved and no drain was placed. The left ureteric catheter was left indwelling for drainage. The port site was closed with vicryl number 1 suture and skin staples were applied. Postoperative recovery was uneventful and per urethral catheter was removed on day 2, and patient was discharged on day 4. Final histopathology confirmed marked interstitial fibrosis, sclerosis, and tubular atrophy with absent glomerular structures suggestive of nonfunctioning kidney.

## 3. Discussion

Urinary tract anomalies account for 3% of all the congenital anomalies. Incidence of ectopic pelvic kidney is 1 in 2500 live births with left kidney being more commonly affected [1, 2]. The ectopic position of kidney is due to the arrest of its ascent during the development [3]. Majority of the ectopic kidneys remain asymptomatic during life and go undetected. It may present with pain, hydronephrosis, and pyelonephritis, renosigmoid fistulae, or lithiasis [4]. Preoperative assessment of the pelvic and vascular anatomy of the ectopic kidney is essential for a successful surgical outcome. Preoperative imaging to delineate anomalous vascular anatomy is essential, and ureteral catheter placement is useful for intraoperative identification purposes. Although urologists are well versed with laparoscopic pelvic procedures, laparoscopic pelvic nephrectomy possesses certain challenges. The first is the presence of anomalous vascular anatomy which often requires extensive dissection to prevent inadvertent injury of vital pelvic vessels. The second is the difficulty in location and dissection of hilar structures due to limited space in the pelvis. The third is the difficulty in port placement due to medially placed pelvic ectopic kidney [2]. Over the past two decades, the field of urology has witnessed a revolution from the era of predominant traditional open surgery to endoscopic and laparoscopic surgical techniques. This revolution has gained widespread acceptance due to well-documented advantages of minimally invasive surgery. Following the introduction of laparoscopy in the field of urology, the number of centers performing this approach has been increasing steadily. It is well accepted that laparoscopy provides surgical outcomes with efficacy equal to that of open surgery [5]. Due to the clear advantages of laparoscopic surgery, the indications of this approach have expanded dramatically over the years [6]. Similarly, the renowned benefits of minimal invasive surgery such as less pain, quicker convalescence, and improved cosmesis are also well perceived by the patients. Moreover, decreased intraoperative blood loss, lesser need of transfusions, and shortened hospital stay make this approach as a “sine qua non” of the current and future urologic surgery. Laparoendoscopic single site surgery (LESS) was first suggested as a consensus nomenclature suggested by the Urologic NOTES Working Group in 2008. Laparoendoscopic single site surgery is now widely accepted as a general term for all new surgical procedures using one skin incision for access of camera and instruments, with or without an additional port of max 5 mm [7]. Advantages of this new approach regarding minimal invasiveness over conventional laparoscopy are in discussion, but they are not yet proven, and cosmesis seems to be driving this technology to a considerable extent [8–10].

We demonstrated in our case that SIMPLE technique is feasible option of nephrectomy for nonfunctioning pelvic kidney in expert laparoscopic surgeon. It is associated with an excellent outcome, minimal invasiveness, and short hospital stay. It has better best aesthetic outcome in patients. Moreover advantages of SIMPLE over LESS and conventional laparoscopy are that there is no need for any additional expenses of special port and instruments during surgery. However more studies are needed to standardize this approach in such cases.
